# Desiccation and rehydration dynamics in the epiphytic resurrection fern *Pleopeltis polypodioides*

**DOI:** 10.1093/plphys/kiab361

**Published:** 2021-08-02

**Authors:** Kyra A Prats, Craig R Brodersen

**Affiliations:** School of the Environment, Yale University, New Haven, Connecticut, USA

## Abstract

The epiphytic resurrection—or desiccation-tolerant (DT)—fern *Pleopeltis polypodioides* can survive extreme desiccation and recover physiological activity within hours of rehydration. Yet, how epiphytic DT ferns coordinate between deterioration and recovery of their hydraulic and photosynthetic systems remains poorly understood. We examined the functional status of the leaf vascular system, chlorophyll fluorescence, and photosynthetic rate during desiccation and rehydration of *P. polypodioides*. Xylem tracheids in the stipe embolized within 3–4 h during dehydration. When the leaf and rhizome received water, tracheids refilled after ∼24 h, which occurred along with dramatic structural changes in the stele. Photosynthetic rate and chlorophyll fluorescence recovered to predesiccation values within 12 h of rehydration, regardless of whether fronds were connected to their rhizome. Our data show that the epiphytic DT fern *P. polypodioides* can utilize foliar water uptake to rehydrate the leaf mesophyll and recover photosynthesis despite a broken hydraulic connection to the rhizome.

## Teaser

After desiccation, an epiphytic resurrection fern recovers photosynthetic activity—whether or not it is connected to the rhizome—while xylem remains embolized.

## Introduction

Desiccation-tolerant (DT) ferns live at the extreme margins of the environmental envelope capable of sustaining plant life. They can temporarily suspend most biological functions when fully dehydrated, reaching water potentials ∼−100 MPa after losing 90% of intercellular water and reaching equilibrium with the surrounding atmosphere (Gaff, 1997). After precipitation events, they rapidly recover to full physiological activity in a matter of hours with no apparent long-term damage. While desiccation tolerance is primarily found in extant bryophytes and pteridophytes (Oliver et al., 2000; Alpert and Oliver, 2002), it is also a trait found in some small angiosperms (Gaff and Oliver, 2013). Around 1% of ferns are considered DT as sporophytes (Proctor and Pence, 2002; Watkins et al., 2007a, 2007b; Pittermann et al., 2013), and DT ferns are often found in xeric habitats that present a number of physiological and reproductive constraints. While our understanding of the physiology of terrestrial DT ferns has improved (Holmlund et al., 2019, 2020), much less is known about epiphytic DT ferns that face a different set of challenges.

The DT and epiphytic Resurrection fern (*Pleopeltis polypodioides* (L.) Andrews and Windham, synonym *Polypodium polypodioides*; Polypodiaceae) is distributed through southeastern North America to South America (GBIF Secretariat, 2019) in humid regions with year-round precipitation (Potts and Penfound, 1948). This species grows as an epiphyte, with a preference for oak branches as its primary substrate in the southern United States (e.g. *Quercus virginiana*; Potts and Penfound, 1948). Early studies attributed this preference to the ability of *Q. virginiana* bark to retain moisture and thus serve as a capacitive reservoir for the rhizome (Pessin, 1925; Potts and Penfound, 1948). In the lab, this species of fern can lose up to 97% of its water content and completely restore leaf water content and recover to predesiccation photosynthetic performance within a few hours (Stuart, 1968; John and Hasenstein, 2017). During the desiccation phase, mesophyll deformation occurs, presumably due to turgor loss, thereby causing the lamina to fold or curl (Pessin, 1924). This structural change in the presentation of the leaf is thought to minimize both mechanical (Helseth and Fischer, 2005; Layton et al., 2010) and photo-oxidative damage (Muslin and Homann, 1992; Alpert, 2000). Recovery of photosynthesis is tightly coupled to both relative water content (RWC) and relative humidity (Stuart, 1968), and established modes of rehydration occur via either the rhizome or by foliar water uptake (John and Hasenstein, 2017). Both pathways have their own temporal dynamics and biochemical and anatomical adaptations, yet rhizome recovery is slower (John and Hasenstein, 2017, 2020).

Previous work in terrestrial DT ferns suggests that xylem embolism occurs in the stipe during the desiccation phase, thereby blocking the vascular pathway between the rhizome and the leaf lamina (Holmlund et al., 2019, 2020). Positive root pressure was found to be important for the recovery of both the Goldback fern (*Pentagramma triangularis*) and the Coffee fern (*Pellaea andromedifolia*), xeric terrestrial resurrection ferns (Holmlund et al., 2020), strongly implicating the rhizome in reestablishing a hydraulic connection that enables restoration of leaf turgor and recovery.

Epiphytic ferns growing in mesic habitats, but with more frequent rainfall events and conditions that favor dew formation, may rely less upon root pressure and the rhizome than do their terrestrial and xeric counterparts. The smaller rhizome mass and dependence on the bark substrate capacitance to meet the transpirational demands of the leaves may be less reliable than foliar inputs. Highly vulnerable stipe xylem could act as a “hydraulic fuse” (Zimmerman, 1983; Tyree et al., 1993; Wolfe et al., 2016), thereby decoupling the leaf from the rhizome, and ensuring long-term plant survival. Under sustained periods of leaf wetness or high relative humidity, conditions could arise that might facilitate embolism removal with (Holmlund et al., 2020) or without (Rolland et al., 2015) a metabolic mechanism like root pressure. Thus, rehydration and restoration of photosynthetic capacity may be possible without any contribution from the rhizome and vascular system, but survival of the rhizome would be critical for long-term survival and growth of new fronds.

One anatomical feature that is crucial for recovery in *P. polypodioides* is the peltate scales on the abaxial (lower) surface of fronds, which are shield-like trichomes made of cellulose and lipids. In ferns within the Polypodiaceae and Anemiaceae, scales have been shown to protect leaf tissue against excess photodamage (Watkins et al., 2006; Farrant et al., 2009). The peltate scales in *P. polypodioides* have been shown to slow dehydration and assist with rehydration via foliar water uptake (Pessin, 1924; Potts and Penfound, 1948; John and Hasenstein, 2017, 2018).

Despite previous work documenting the decline and recovery of both water content and photosynthesis in *P. polypodioides* (Pessin, 1924; Stuart, 1968; John and Hasenstein, 2017, 2018), the exact timing and coordination between loss of hydraulic conductivity between the rhizome and the leaf and declines in photosynthetic capacity are not known. Given the tight coupling between relative humidity and RWC of the leaf, and the limited capacity for water storage in the bark substrate and rhizome, high evaporative demand could quickly exceed the liquid water reservoirs available to sustain both mesophyll turgor and photosynthesis. Furthermore, extended evaporation of water from the leaf could cause substantial dehydration of the rhizome and threaten the long-term survival of the plant. Yet, this species persists and thrives in epiphytic environments.

Here, we examined the coordination between the functional status of hydraulic and photosynthetic systems that occurs during desiccation and recovery of the epiphytic resurrection fern *P. polypodioides*. We aimed to (1) determine whether losses of conductivity in the xylem due to embolism influenced photosynthetic capacity at different stages of dehydration and (2) observe the temporal dynamics of photosynthetic recovery and determine its dependence on the functional status of the xylem, or whether recovery can occur without a connection to the rhizome and root system. During the recovery phase, not only do the leaves need to restore turgor and photosynthetic capacity, but they also need a supply of water to sustain transpiration. If we observe similar timing of photosynthetic and hydraulic decline and recovery, then photosynthetic capacity is closely linked to the functional status of the xylem. On the other hand, if fronds without connection to the rhizome can recover photosynthetic capacity along a similar timeframe as fronds that are connected to the rhizome, then foliar water uptake could be the primary pathway of rehydration. Our study combines the use of multiple noninvasive techniques—including high-resolution X-ray computed tomography (microCT), chlorophyll fluorescence imaging, and gas exchange—to capture many previously unknown facets of desiccation and recovery dynamics. Given that ferns represent a critical evolutionary link between nonvascular plants and seed plants, studying resurrection dynamics in ferns—and accounting for any differences between terrestrial and epiphytic DT ferns—can inform our understanding of how resurrection dynamics differ across the phylogeny.

## Results

### Brightfield microscopy

**Figure 1:**
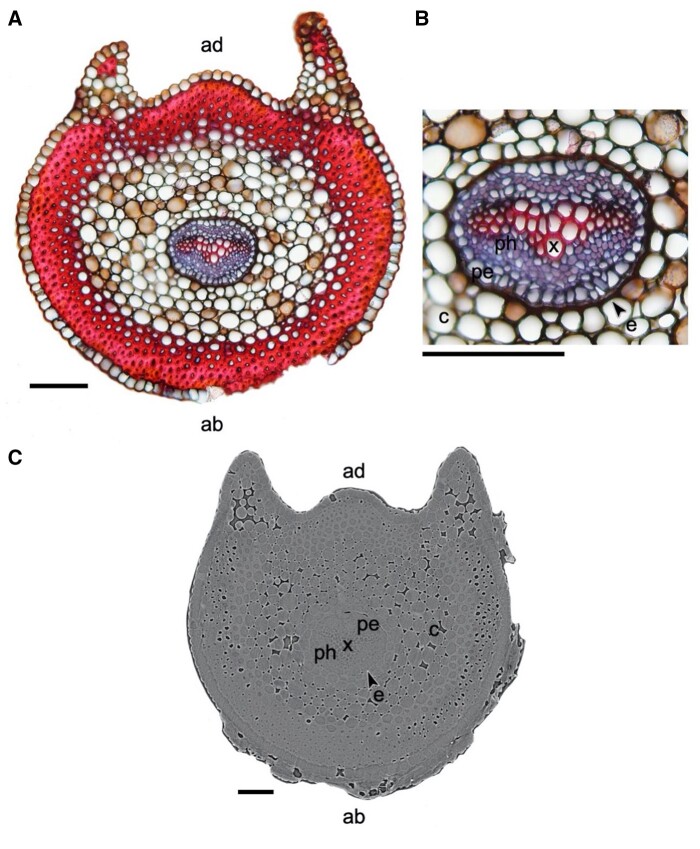
*Pleopeltis polypodioides* transverse stipe anatomy from light microscopy and X-ray microCT imaging. A and B, Light micrographs of a freehand stipe cross-section at two magnifications, with a combination of Safranin (red) and Astrablue (blue) stains. Higher magnification of the stele (B) shows the boundary between the endodermis (black arrow) and the cortex where separation of those tissues was observed during dehydration. C, MicroCT image of well-hydrated stipe cross section. Labeling in (A) and (C) show the adaxial (ad) and abaxial (ab) sides of the stipes, while labeling in (B) and (C) show the xylem (x), phloem (ph), pericycle (pe), endodermis (e) with black arrows, and cortex (c) . Scale bars = 0.5 mm in (A and B), 0.1 mm in (C).

The freehand stipe cross sections under both well hydrated (Figure 1;Supplemental Figure S1A) and dehydrated (Supplemental Figure S1B) conditions provide context for the grayscale X-ray microCT images of the stipe. The dehydrated cross section (Supplemental Figure S1B) shows that a physical separation of the vascular bundle occurs between the endodermis and the surrounding cortex cells. During dehydration, most of the cell collapse likely occurs in the pericycle tissue just inside the endodermis and in some of the phloem. We found no evidence for tracheid collapse or deformation during the dehydration process in the light microscopy images.

### Characterizing and visualizing dehydration

Time-lapse imaging of the fronds during dehydration in the lab was consistent with previously documented changes in leaf shape (Supplemental Movies S1–S3). Leaves in a well-hydrated state were pliable and dark green on both surfaces. As the leaves dried, the pinnae folded with concave curvature toward the adaxial surface, and the rachis began to curl inward simultaneously (Supplemental Movie S1). In the desiccated state, the leaf remained curled, with only the adaxial surface facing outward. After 24 h the leaves became brittle and were difficult to uncurl without cracking and tearing of the lamina.

**Figure 2:**
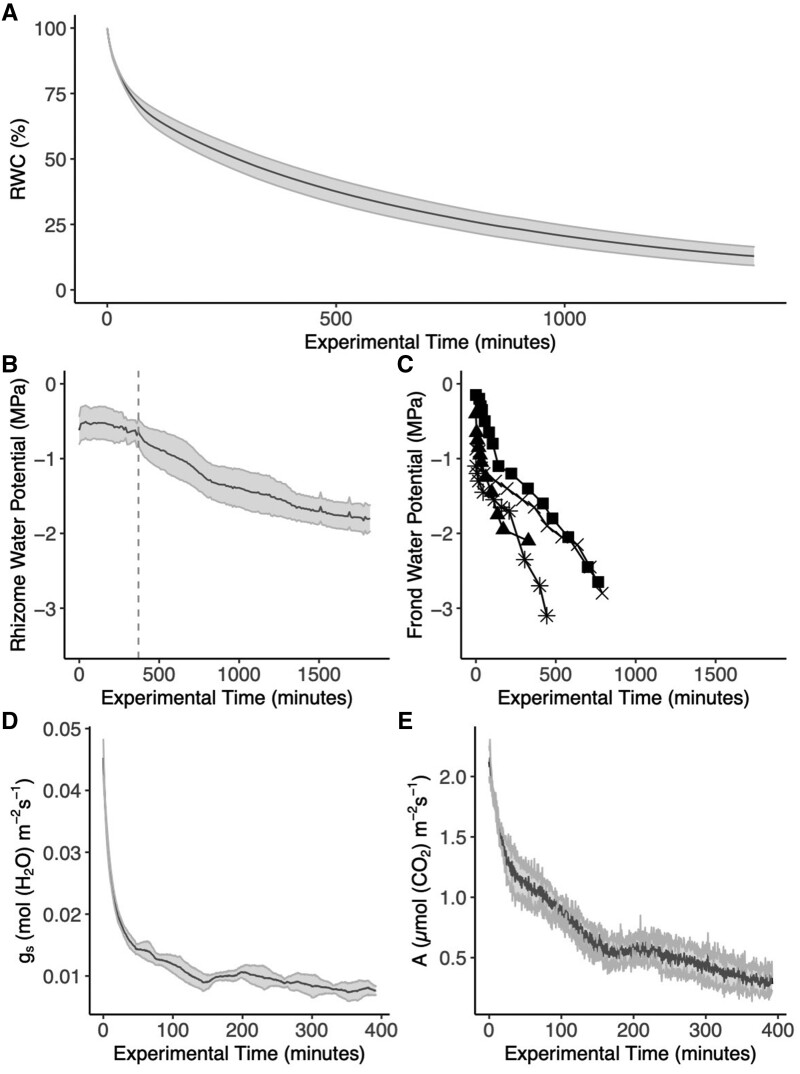
Physiological changes of *P. polypodioides* stipes during dehydration. A, Average RWC (%) of frond and rhizome material (*n* = 5) over a 24-h dehydration (dark gray line). At 24 h, the average RWC was 14% (±7.26 SD). Light gray represents standard error. B, Rhizome water potentials (*n* = 8) while well-hydrated (left of the dashed line). A 24-h dehydration began at the vertical dashed line. The dark gray line represents the average, while light gray represents standard error. C, Frond water potentials (*n* = 4, different shapes) measured with a pressure chamber over the course of dehydrating for a maximum of 13 h. D, Stomatal conductance of fronds (*n* = 3) over a 6-h dehydration. The dark gray line represents the average, and light gray represents standard error. E, Photosynthetic rate (*A*) of fronds (same as in (D)) over a 6-h dehydration. The dark gray line represents the average, and light gray represents standard error. Note the differing *x*-axis scales across the panels.

During the first 24 h of bench dehydration, the RWC of frond and rhizome material declined steeply at first and then more slowly (Figure 2A), with a final mean RWC of 14.1% (±7.26 standard deviation (sd)) (*n* = 5). Fronds that were dehydrated for 24 h in our subsequent experiments were assumed to fall within this average range of RWC.

Mean rhizome water potentials measured with thermocouple stem psychrometers were −0.54 (±0.03 sd) MPa under well-watered steady state conditions before the experimental dry down (Figure 2B). Rhizome water potentials then declined to −1.80 (±0.42 sd) MPa over the 24- h desiccation period. All plants showed a steep initial decline in rhizome water potential over the first 4 h, followed by a slower decline. Given that the rhizome was tightly sealed in paraffin film during the dry-down, we note that the rate of decline and absolute maximum and minimum water potentials are likely conservative estimates. Frond water potentials measured over time under identical conditions with the pressure chamber showed similar declines over the same period (Figure 2C).

Gas exchange declined along with frond water status during a shorter, 6 h dehydration. Both stomatal conductance (Figure 2D) and photosynthetic rate (Figure 2E) initially declined sharply, and declined more slowly after 2–3 h.

A limited set of transverse X-ray microCT cross sections of pinnae in a well-hydrated state were compared to those in a dehydrated state (Supplemental Figure S2). Mesophyll cells were turgid, and the leaf tissue was fully expanded in the well-hydrated state (Supplemental Figure S2A). In contrast, mesophyll cells from pinnae in the dehydrated state had clearly lost turgor and appeared shrunken in comparison (Supplemental Figure S2B).

Pressure-volume curves revealed that the average frond water potential at the turgor loss point was −1.35 (±0.31 sd) MPa (*n* = 6). These data, along with our time-lapse imaging and microCT imaging of the leaves, suggest that cell turgor is lost within the initial 3–4 h of the dry-down experiments as the leaves begin to curl.

### Dynamics of xylem embolism formation and recovery: microCT

**Figure 3:**
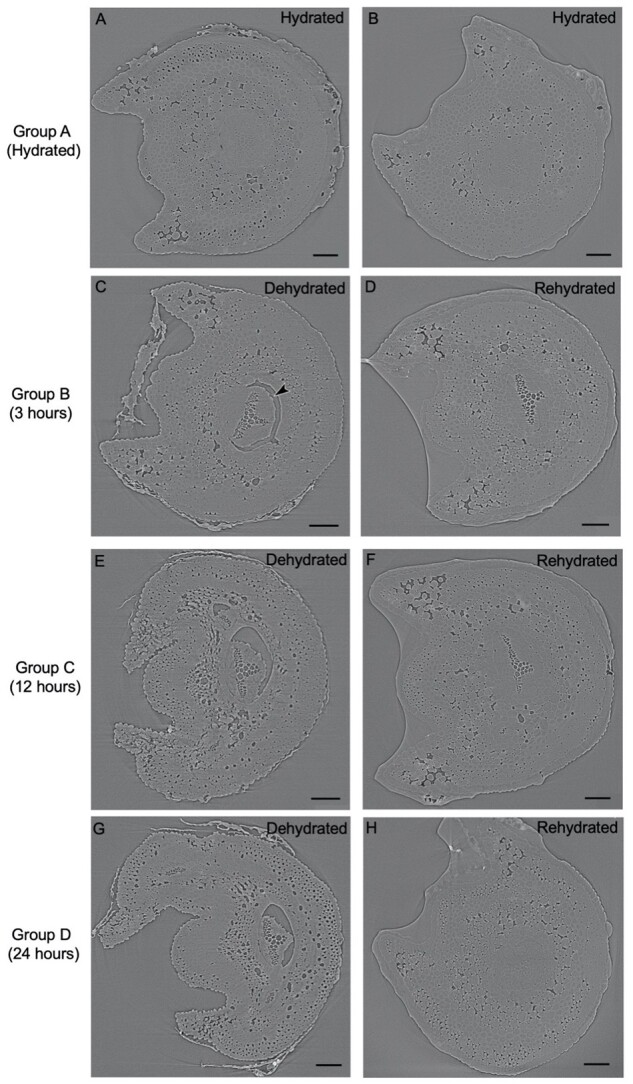
Dehydration and rehydration of *P. polypodioides* stipes observed with X-ray microCT imaging. A–H, MicroCT scans of stipes in each group. The xylem is water-filled and the cortex is hydrated in the Group A scans ((A) and (B)). Xylem embolism forms after dehydration in (C), (E), and (G), and persists in (D) and (F). Repair of embolism is only seen in (H), after 24 h of rehydration. Lacunae—gaps between the vascular bundle (stele) and the endodermis are seen in (C) (noted with an arrow), (E), and (G). All scale bars = 0.1 mm.

Embolism was visible in stipes attached to rhizome sections after just 3 h (Group B) of dehydration (Figure 3), and was also present at 12 h (Group C) and 24 h (Group D) of dehydration. After 3 h of dehydration, the stele began to shrink from the surrounding endodermis, with an air-filled gap or lacuna between the endodermis and the stele, always forming on the abaxial side of the stipe. In general, these gaps became bigger and more pronounced after 12 and 24 h of dehydration. The stipe also began to shrink and curl with dehydration. Rehydration of the stipe and frond also occurred quickly, and microCT imaging revealed the recovery of stipe shape and the disappearance of large gaps between the stele and endodermis. However, tracheids remained embolized after 3 and 12 h of rehydration, and embolism repair was only apparent after 24 h of rehydration (Figure 3).

**Figure 4:**
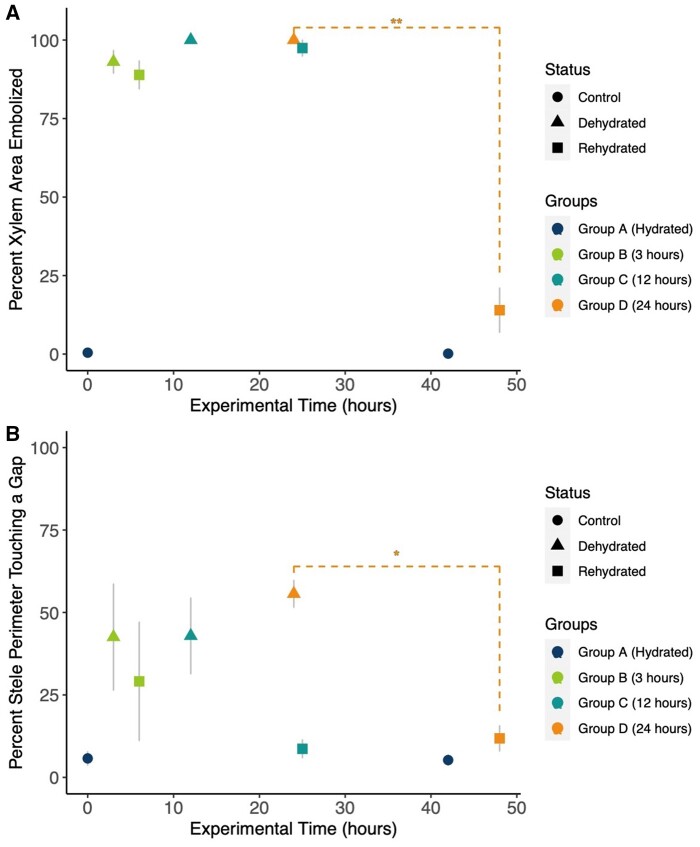
Percent area embolized and percent of the stele perimeter touching a gap from X-ray microCT images. A, Mean percent area embolized in each group over time; Group A is the hydrated control group, Group B is the 3-h group, Group C is the 12-h group, and Group D is the 24-h group (*n* = 3 per group). A repeated measures ANOVA showed that the percent xylem area embolized was significantly different for the experimental groupings across time (*F*(7, 16) = 200.525, *P* < 0.001), but this was driven by the differences between the 24-h dehydrated and 24-h rehydrated Group D (*P* = 0.007, **Bonferroni correction). Gray bars represent standard error of the mean. B, Mean percent of the stele perimeter touching a gap (lacuna) between the endodermis and the vascular bundle over time; Group A is the hydrated control group, Group B is the 3-h group, Group C is the 12-h group, and Group D is the 24-h group. The repeated measures ANOVA revealed that the percent of the stele perimeter touching a gap was significantly different between treatment groups across time (*F*(7, 16) = 4.2, *P* = 0.008), but this was driven by the pairwise differences in Group D between the 24-h dehydrated and 24-h rehydrated values (*P* = 0.019, *Bonferroni correction). Gray bars represent standard error of the mean.

Within 3 h of dehydration, 93.1% (±6.3 sd) of the xylem area was embolized (Figure 3). Percent embolism increased to 100% after 12 and 24 h of dehydration (Figure 4A). Xylem refilling was minimal and not statistically significant after 3 and 12 h of rehydration, but refilling was substantial after 24 h of rehydration (Figure 4A). Percent xylem area embolized was significantly lower within the experimental groupings across experimental time (repeated measures analysis of variance (ANOVA), *F*(7, 16) = 200.525, *P* < 0.001). However, post-hoc analyses with a Bonferroni correction revealed that only the pairwise difference within Group D (i.e. between the 24 h dehydrated and rehydrated scans) was statistically significantly different (*P* = 0.007). The percent of xylem area embolized was negligible for the hydrated control during both measurements.

We then quantified the physical changes to the stele resulting from dehydration and rehydration by measuring the size of the lacunae that formed between the endodermis and the surrounding cortex. After 3 h of dehydration, large lacunae developed between the stele and the cortex, and the percent of the stele perimeter exposed to a lacuna rather than having direct contact with the cortex was 42.5% (±27.9 sd) (Figure 4B). The extent of the lacunae remained unchanged for 12 h of dehydration (42.9% ±19.9 sd), and increased to 55.6% (±7.1 sd) after 24 h of dehydration, that is, over half of the stele separated from the surrounding cortex as the stipes dehydrated. Unlike in *Selaginella* (Cardoso et al., 2020), we did not observe any trabeculae, endodermal cells that connect the cortex with the pericycle and the vasculature. Lacunae quickly disappeared as the pericycle and endodermis swelled to meet the cortex cells during the rehydration (Figures 3 and 4). Three hours of rehydration resulted in a 13% decrease in the percent of the stele perimeter touching a gap. Lacunae nearly completely disappeared after 12 and 24 h of rehydration, where only 8.6% (±4.7 sd) and 11.8% (±6.6 sd) of the stele perimeter was in contact with a lacunae gap, respectively. The percentage of stele perimeter touching a gap was statistically different for the experimental groupings across time (repeated measures ANOVA, *F*(7, 16)  = 4.2, *P* = 0.008). Post-hoc analyses with a Bonferroni adjustment revealed that only the pairwise difference between the 24-h dehydrated and 24-h rehydrated group was significantly different (*P* = 0.019).

Using the microCT images, we also measured the diameter of the tracheids that were air versus water-filled at the different time points to determine if there was a relationship between tracheid diameter and vulnerability to cavitation. The mean tracheid diameter was 7.8 µm (±4.1 sd) (Supplemental Figure S3A). We measured the difference in tracheid diameters between embolized and water-filled conduits using nine reconstructions that had a mix of embolized and water-filled tracheids (all three reconstructions from the hydrated control group, 3-h dehydrated group, and 24-h rehydrated group). To test if embolized tracheids were a different size from nonembolized tracheids, we performed *t* tests on tracheid diameter within the 3 h dehydrated group and within the 24-h rehydrated group. The average diameter of embolized tracheids was significantly larger than that of water-filled tracheids in both groups; for the 3 h dehydrated group, the mean diameter of embolized tracheids was 8.0 µm (±4.4 sd, *n* = 251), while the mean diameter of nonembolized tracheids was 5.6 µm (±2.5 sd, *n* = 26; *t* = −4.3, df = 43.6, *P* = 0.00009; Supplemental Figure S3B). Similarly, in the 24-h rehydrated group, the mean diameter of embolized tracheids was 14.0 µm (±4.4 sd, *n* = 21), while the mean diameter of nonembolized tracheids was 7.5 µm (±3.3 sd, *n* = 254; *t* = −6.6, df = 21.8, *P* = 0.000001; Supplemental Figure S3C). It is important to note that the tracheid sample size between embolized and nonembolized tracheids for both tests was not equal.

### Chlorophyll fluorescence

**Figure 5:**
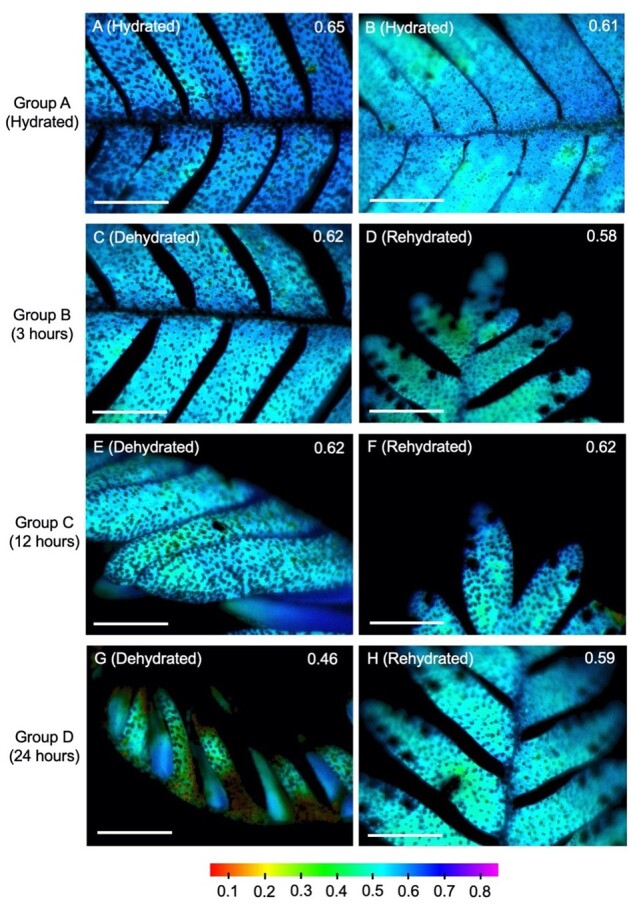
Chlorophyll fluorescence imaging of *P. polypodioides* leaves at different stages of dehydration and rehydration. Representative *F_v_′/F_m_′* images for each experimental group appear in (A–H). Here, the abaxial surface of fronds was imaged. Values in the upper right corner of each image represent the average *F_v_′/F_m_′* of 10 AOIs of abaxial leaf tissue for that image. The false-color scale is based on the measured *F_v_′/F_m_′* data, where healthy leaves for this species are typically around 0.65–0.70 (blue), and areas of lower photosynthetic capacity are shown in green and yellow. All scale bars = 5 mm.

**Figure 6:**
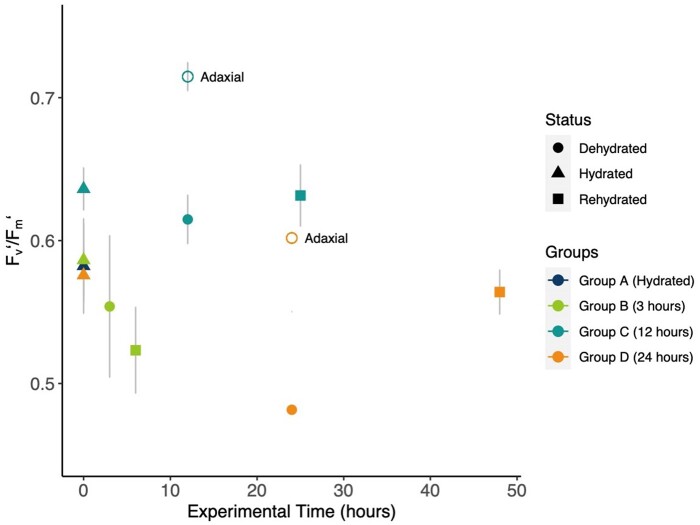
Changes in *P. polypodioides* leaf chlorophyll fluorescence in response to dehydration and rehydration. Shown are the abaxial *F_v_′/F_m_′* (solid shapes) and adaxial *F_v_′/F_m_′* (open shapes) from fluorescence imaging across time. Colors correspond to the experimental group (*n* = 3 per group), while shapes represent the hydration status. At 12 and 24 h of dehydration, the adaxial surface became visible in some of the images due to leaf curling. The dehydrated adaxial *F_v_′/F_m_′* (open circles) was higher than the dehydrated abaxial values. Gray bars show the standard error of the mean.

Well-hydrated abaxial *F_v_′/F_m_′* measurements on leaves used for the microCT imaging revealed that mean abaxial *F_v_′/F_m_′* was between 0.58 and 0.63 for all treatment groups at the start of the experiment (Figure 5A). The *F_v_′/F_m_′* response to dehydration and rehydration varied across treatments, with *F_v_′/F_m_′* further declining after 3 h of dehydration and 3 h of rehydration (Group B) but remaining relatively constant after 12 h of dehydration and 12 h of rehydration in Group C (Figure 5, C–H). Substantial decline during dehydration and recovery to hydrated values was observed for the 24-h treatment Group D (Figure 5H). The representative *F_v_′/F_m_′* images capture the physical curling of the leaf material after 12 and 24 h of dehydration (Figure 5, E and G, respectively). Within these images, it is also apparent that while the abaxial side of the frond was exposed and experienced a decrease in *F_v_′/F_m_′* during dehydration, the adaxial side of the frond appeared protected and adaxial dehydrated *F_v_′/F_m_′* measured higher than abaxial dehydrated values (Figure 6). The average abaxial *F_v_′/F_m_′* was not statistically different across the groups (repeated measures ANOVA, *F*(2, 11) = 3.6, *P* = 0.06).

Since only abaxial frond tissue was imaged at the beamline, additional experimentation comparing adaxial and abaxial *F_v_′/F_m_′* imaged values was conducted (Supplemental Figure S4, A–D). From these follow-up experiments, the *F_v_′/*F_m_*′* of abaxial frond surfaces was shown to be consistently lower than that of adaxial frond surfaces, both in well-hydrated and 24-h dehydrated states (*F*(3, 16) = 14.9, *P* < 0.0001; Supplemental Figure S4E). The post-hoc analysis with a Bonferroni adjustment showed that adaxial and abaxial *F_v_′/F_m_′* were significantly different when well-hydrated (*P* = 0.038) and after 24 h of dehydration (*P* = 0.002). We found that the peltate scales did not account for the difference in abaxial and adaxial *F_v_′/F_m_′*, as experimental removal of the peltate scales did not lead to any difference in the abaxial values (Supplemental Figure S4F). The ANOVA comparing abaxial *F_v_′/F_m_′* with peltate scales, abaxial *F_v_′/F_m_′* without peltate scales, and adaxial *F_v_′/F_m_′* was significant (*F* = 11.16, *P* = 0.002). Post-hoc analysis with a Bonferroni adjustment confirmed that the adaxial mean was significantly different from both abaxial means with and without peltate scales (*P* = 0.002 and P = 0.014, respectively), while there was no significant difference between the abaxial means (*P* = 0.98).

### Visualizing dehydration and rehydration: frond-only and rhizome-only rehydration

Time-lapse images of detached fronds during benchtop dehydration and subsequent rehydration illustrated that detachment from the rhizome did not prevent fronds from rehydrating. Fronds began rehydrating immediately, and leaf tissue unfurled completely within 12 h. When in the presence of moisture and maintained in a humid environment, these detached fronds remained green for 3 months despite being disconnected from the rhizome (Supplemental Movie S2).

Time-lapse images of the rhizome-only rehydration show that half of the fronds unfurled completely within 18–23 h after rehydration (Supplemental Movie S3). This is consistent with results from John and Hasenstein (2017), which found that fronds rehydrated via the rhizome recovered within 12 h. Half of the fronds in our rhizome-only rehydration did not recover fully within the 36 h of rehydration.

### Photosynthesis, frond detachment, and recovery

**Figure 7:**
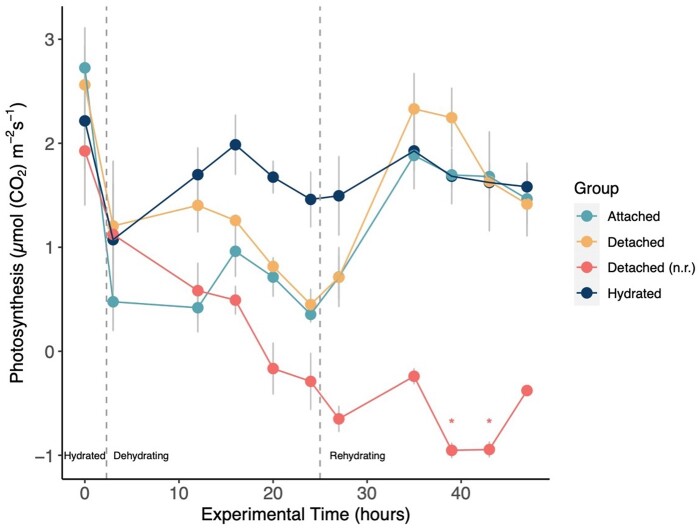
Photosynthetic response of *P. polypodioides* leaves to dehydration and rehydration. See Supplemental Table S1 for description of the different treatments. The repeated measures ANOVA was significant (*F*(10, 121) = 3.654, *P* = 0.0003). Post-hoc Bonferroni corrections revealed that at 39 h (16 h of dehydration), the photosynthetic rates of the Attached group and the detached group were statistically different from that of the detached and not rehydrated group (*P* = 0.028 and *P* = 0.029, respectively, *). Additionally, at 43 h (20 h of rehydration), the photosynthetic rates of the Attached group and the hydrated control group were statistically different from the photosynthetic rate of the detached (n.r.) group (*P* = 0.037 and *P* = 0.039, respectively, *). The photosynthetic rates of the Attached group and the detached group were not significantly different at any time point during dehydration or rehydration. Gray bars show the standard error of the mean, *n* = 3 per group. Gray dashed lines mark the onset of dehydration and then rehydration.

Well-hydrated photosynthetic rates were comparable across all groups before beginning the dehydration treatments (Figure 7). Differences between the photosynthetic rates of each treatment group began to emerge during dehydration. By 24 h of dehydration, the photosynthetic rate of the three dehydrating groups declined by an average of 88% compared to the hydrated control group. Upon rehydrating the attached and detached fronds from the Attached group and the detached group, respectively, within 8 h of rehydration, both groups recovered to photosynthetic rates comparable to—and even surpassing—the photosynthetic rates of the Hydrated control group (Figure 7). The net photosynthetic rate of the detached and not rehydrated (n.r) group remained below zero for the last half of the experiment and showed no recovery.

The photosynthetic rates were significantly different between treatment groups across the experimental time points (repeated measured ANOVA, *F*(10, 121) = 3.654, *P* = 0.0003). However, post-hoc analyses with a Bonferroni adjustment revealed that the pairwise differences between groups at specific time points were only statistically significantly different at 39 h (16 h rehydrated) and at 43 h (20 h rehydrated) of the experiment (Figure 7, asterisks). Specifically, at 39 h, the Attached group had experienced 16 h of rehydration and the photosynthetic rate was statistically different from that of the detached and n.r group (*P*= 0.028). The detached group at 16 h of rehydration also had a photosynthetic rate that was statistically different from that of the detached and n.r group (*P* = 0.029). Similarly, at 43 h into the experiment, the photosynthetic rate of the Attached group—which had been rehydrating for 20 h—was statistically different from the photosynthetic rate of the detached and n.r group (*P* = 0.037). Finally, the photosynthetic rate of the detached and n.r group at 43 h of dehydration was statistically different from the photosynthetic rate of the Hydrated control group (*P* = 0.039). There were no statistically significant differences between the photosynthetic rates of the attached and the detached groups during any stage of dehydration or rehydration.

### Fluorescence microscopy for foliar water uptake

**Figure 8:**
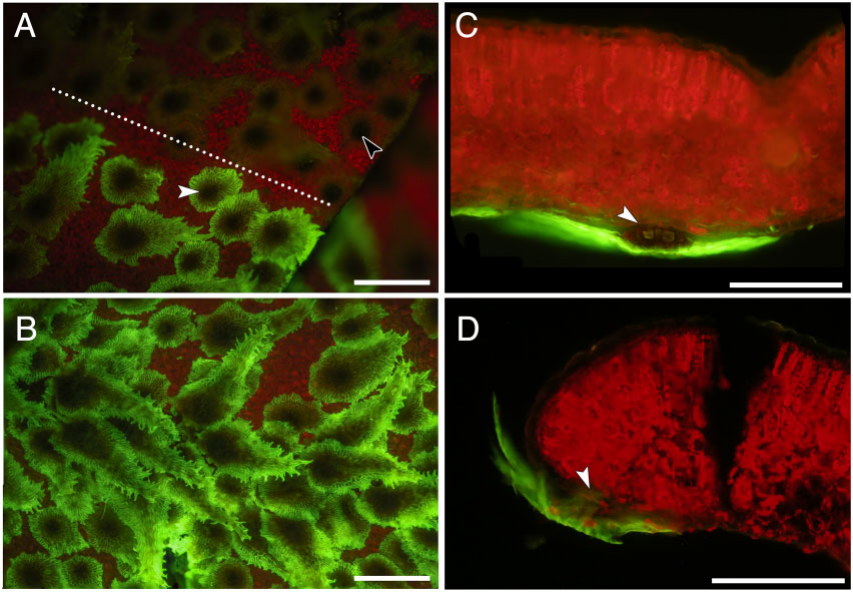
Fluorescence microscopy images of peltate scales on the abaxial surface of *P. polypodioides*. A, Abaxial leaf surface at the LY dye droplet boundary (dotted line). Scales exposed to the droplet show dye uptake (white arrow), while scales with no dye contact only show faint green autofluorescence (black arrow). Red chlorophyll autofluorescence emitted from the mesophyll is visible in the background. B, A second abaxial surface view of the peltate scales showing uptake of the fluorescent LY dye. C and D, Transverse cross-sections through representative leaves showing LY fluorescence within the peltate scales, but with no dye movement into the mesophyll after 30 min. White arrows show where the stalk of the peltate scales meets the epidermis. All scale bars =500 µm.

Upon application of foliar water uptake dye tracer Lucifer Yellow (LY) to the abaxial surface of the leaf, uptake of the dye was clearly visible within 15 min of application and uptake was isolated to the peltate scales (Figure 8). Peltate scales and epidermal cells adjacent to the applied droplet of the dye showed no uptake (Figure 8A). Transverse sections of the leaves 30 min after the removal of the dye from the epidermis showed the presence of dye in the scales, but it did not enter the mesophyll (Figure 8, C and D). These patterns were consistent across the six leaves included in the dye uptake experiment.

## Discussion

Our data show that epiphytic *P. polypodioides* experienced complete hydraulic failure of the stipe xylem during the dehydration period, but over the same period lost only ∼50% of its photosynthetic rate, and 10%–20% of its photosynthetic capacity, which we estimated using chlorophyll fluorescence as a proxy. The highly vulnerable stipe xylem and rapid decrease in water potential during the dehydration period was coordinated with rapid loss of water from the mesophyll, where the mean turgor loss point was −1.35 (±0.31 sd) MPa. This sequence of events hydraulically decouples the frond and rhizome tissues, thereby preventing excessively negative water potentials in the rhizome. This hydraulic connection then remained broken until both the rhizome and leaf were fully saturated for at least 24 h. Recovery of leaf shape, turgor, and photosynthesis was much more rapid, which strongly suggests that foliar rehydration can both provide sufficient water for rehydration and also sustain photosynthesis for some period of time. Unlike terrestrial DT ferns *P. triangularis* and *P. andromedifolia* (Holmlund et al., 2020), *P. polypodioides* does not rely on a hydraulic connection to the rhizome or roots for rehydration and recovery, and can completely recover photosynthetic capacity and gas exchange via foliar water uptake under laboratory conditions. The fronds are resilient and continue to photosynthesize independent of the rhizome in the presence of high relative humidity and a surface film of water to support foliar water uptake. Overall, this strategy works well for an epiphyte growing in regions that receive frequent precipitation, as these plants will rely on frequent wetting events or dew formation for their recovery.

### Dynamics of frond dehydration

The average RWC after 24 h of dehydration (14.1% ±7.26 sd) revealed that these ferns lose a large percentage of their water content over the course of the dry-downs, with a steep initial decline followed by slow, steady decline (Figure 2). This rapid decline of RWC is likely the driver of turgor loss and declining frond and rhizome water potential during the same period. Fronds seemed to reach more negative water potential values more quickly than the rhizomes, which potentially highlight the vulnerability of fronds compared to rhizomes; one caveat is that rhizome water potentials are likely conservative estimates given that rhizomes were wrapped with parafilm to secure stem psychrometers. With declines in water status, stomatal conductance and photosynthetic rate decreased as stomata closed. Coupled with our microCT results, our data show that after 3 h of dehydration, the fronds reached their turgor loss point, RWC was 58.1% (±4.1 sd), and the stipe tracheids were completely embolized. While it is important to note that the relative humidity varied between experiments depending on the facility (see “Materials and methods” for relative humidity values), we still observed that the timing of the decline of the photosynthetic and hydraulic systems remains coupled during dehydration.

### Dynamics of xylem embolism formation and recovery

Nearly all of the tracheids in the stipes were embolized after 3 h of desiccation, and embolism persisted until rehydration occurred for 24 h (Figure 3). These results illustrate that the stipe xylem of *P. polypodioides* is quick to embolize, yet slow to repair. Because each fern in each group in the timeline was scanned only once during dehydration and once during rehydration, it is possible that the xylem from the groups that were dehydrated and rehydrated for a shorter amount of time (e.g. the 3 h Group B) were able to repair embolism on a faster timescale than 24 h. However, we scanned each fern only twice to minimize potential artifacts produced by the X-ray imaging system. Given that the resurrection process occurs within 3–4 h in the natural environment, and refilling here required leaf saturation for 12–24 h, our data suggest that it is unlikely that photosynthetic recovery and leaf rehydration is supported primarily by water conduction through the xylem. Instead, our data support the idea of foliar rehydration as the primary means of recovery that facilitates subsequent embolism repair.

Embolism repair is likely possible by at least two nonmutually exclusive mechanisms, which include both passive (capillary action; Rolland et al., 2015) and active modes (Zwieniecki and Holbrook, 2009; Brodersen et al., 2010; Brodersen and McElrone, 2013), as well as a specific suite of anatomical traits and environmental conditions that facilitate the process. Pittermann et al. (2013) speculated that ferns, with their vascular bundles surrounded by parenchyma and cortex cells, might possess the appropriate anatomical organization to support active refilling (Pittermann et al., 2013), despite potential confinement from the endodermis. Once immersed in water, as in our experiment that simulated a heavy rainfall event, xylem water potentials would approach 0 MPa, thereby allowing embolism repair via capillary action. This phenomenon was documented in intact xylem, where gas bubbles dissolved within 30–90 s under the right conditions (Rolland et al., 2015). We did not observe any water droplets on the tracheid walls as seen in actively refilling grapevine stems (Brodersen et al., 2010, 2018), but our sampling frequency and single scan of each stipe limited our ability to track the functional status of the xylem of our plants over time. However, our experimental design provided ideal conditions for refilling to occur via capillarity, that is, a passive rather than active mechanism.

The mechanisms of embolism repair in ferns have only recently been studied, with a focus on terrestrial DT ferns (Holmlund et al., 2019, 2020). Terrestrial DT ferns *P. triangularis* and *P. andromedifolia* were found to employ root pressure to achieve full, whole-plant rehydration following desiccation (Holmlund et al., 2020). This study also proposed that these terrestrial DT ferns likely relied on a combination of root pressure, capillary action, and foliar water uptake during recovery (Holmlund et al., 2020). Notably, our results in the epiphytic *P. polypodioides* show that fronds can recover photosynthetic function during rehydration via foliar water uptake, regardless of whether they are attached to the rhizome and roots (Figure 7). These data point to *P. polypodioides* relying heavily on foliar water uptake, and a physical connection to the rhizome is not necessary for short-term recovery as long as there is a high relative humidity and water available to the leaf surface.

The formation of air-filled gaps between the vascular bundles and the endodermis and cortex also occurred rapidly, but these gaps disappeared in every rehydrated stipe that we scanned, no matter how long the rehydration period. We do not know from the microCT scans the fate of the air upon repair of lacunae, but there is likely enough space within the cortex to accommodate the air volume. Air-filled lacunae formation between the vasculature and the endodermis has been observed in DT fern *P. triangularis* (Holmlund et al., 2019), as well as in spikemoss lycophytes *Selaginella haematodes* and *Selaginella pulcherrima* (Cardoso et al., 2020) and DT angiosperms *Ramonda serbica* and *Ramonda nathaliae* (Rakić et al., 2017). MicroCT images of *Selaginella* revealed that a decline in shoot hydraulic conductance coincided with lacuna formation, and that the lacuna formed before the xylem embolized (Cardoso et al., 2020). These images also revealed that these gaps formed equally around all sides of the vascular bundle (but bundle tissue maintained some connections to the cortex tissue through spread out trabeculae (Cardoso et al., 2020)). Our microCT images of *P. polypodioides* revealed that air-filled gaps were oriented toward the abaxial side of the stipes, with the bundles maintaining connection on the adaxial side of the stipe. This directionality of gap formation is possibly related to the direction of leaf and stipe curling during dehydration, as the adaxial side of the leaf curls inward for protection while the abaxial side is exposed. Collectively, these data suggest that the evaporative demand from the pinnae exceeds the supply provided by the vascular tissue and surrounding capacitive cortex cells, leading to cavitation in nearly all of the tracheids, as well as a depletion of the water storage located in the cortex cells of the stipe. In this species, the endodermis appears to be remarkably rigid, with most of the cellular deformation inside the endodermis occurring in the pericycle adjacent to the xylem (Figure 3).

### Chlorophyll fluorescence

The *F_v_′/F_m_′* images from the beamline were not substantially different in Group B (3 h) and Group C (12 h) while they were dehydrated and rehydrated, but abaxial *F_v_′/F_m_′* declined and recovered substantially during the 24-h dehydration and rehydration cycle of Group D (Figures 5 and 6). In contrast to terrestrial DT ferns where the dark-adapted *F_v_/F_m_* approached zero after 7 months of desiccation (Holmlund et al., 2020), *P. polypodioides* experienced more moderate *F_v_′/F_m_′* values after 24 h of desiccation. In their natural environment, these ferns receive frequent rainfall and do not experience a pronounced dry season (Potts and Penfound, 1948); with frequent precipitation, *P. polypodioides* may need to maintain *F_v_′/F_m_′* to take advantage of rapid rehydration. Adaxial leaf tissue that was exposed in the images did not experience a decline in *F_v_′/F_m_′* during dehydration, perhaps due to the fact that the adaxial leaf surface is protected by leaf curling. Protection of the adaxial side of the leaf, which contains most of the chloroplasts in the palisade layer, through curling may help to protect the chloroplasts so that they may ramp up quickly during recovery.

Fluorescence imaging of both leaf surfaces revealed that *F_v_′/F_m_′* on the abaxial leaf surface was consistently lower than measurements of the adaxial surface. This was true in both well-hydrated and desiccated states. Previous work has shown differences between adaxial and abaxial chlorophyll fluorescence, hypothesizing that differences in chloroplasts between the abaxial and the adaxial leaf surfaces were responsible for the differing fluorescence responses, rather than leaf morphological differences between the palisade and spongy mesophyll (Schreiber et al., 1977). However, other studies have shown that the palisade leaf morphology and high chlorophyll content of adaxial leaf surfaces allow for reabsorption of chlorophyll fluorescence signals, causing the fluorescence to be lower on the adaxial surface than on the abaxial surface (Lichtenthaler et al., 2005). When calculating the *F_v_/F_m_* ratio, this phenomenon translates into higher adaxial *F_v_/F_m_* and lower abaxial *F_v_/F_m_* (Lichtenthaler et al., 2005), as was found in our measurements of *P. polypodioides*. Measurement of adaxial fluorescence may only capture chloroplast function in the palisade parenchyma leaf layers (Vogelmann and Evans, 2002; Kalaji et al., 2014), and it has been proposed that to understand the full picture of leaf fluorescence dynamics, abaxial fluorescence should be taken into account as well (Lichtenthaler et al., 2005). It is important to note that chlorophyll fluorescence measurements do not require a flux of water in the leaf tissue, and thus do not provide information about the true extent of the leaf tissue water status.

### Rehydration, photosynthesis, and foliar water uptake

Fronds recovered to a fully expanded state within 12 h of the frond-only rehydration (Supplemental Movie S2), compared to within 18–23 h during rhizome-only rehydration (Supplemental Movie S3). However, there was a high degree of variability with our rhizome-only rehydration, as half of the fronds did not fully recover within the 36 h of rehydration. The fact that some fronds did not recover may be due to the dry atmospheric conditions in the lab—where evaporation from the leaves exceeded the supply of water from the rhizome—or perhaps due to some variability between fronds in the amount of embolism in the xylem. Overall, our results support those from John and Hasenstein (2017), which reported that rehydration was much faster via frond tissue than through the rhizomes.

Our gas exchange data show that the photosynthetic rates in Groups B and C recovered to predehydration levels after 12 h of rehydration, regardless of whether or not the fronds were attached to the rhizomes. This time frame for recovery is also consistent with our time-lapse imagery (Supplemental Movie S2). Additionally, the recovery of photosynthesis during rehydration occurred before xylem embolism repair in these ferns, highlighting how the xylem and the photosynthetic machinery are largely decoupled during rehydration. These photosynthesis results, together with the xylem embolism recovery results, indicate that functional, water-filled xylem in the stipe is not necessary for ramping up physiological function, as the xylem is slow to repair during rehydration. Instead, foliar water uptake supplies water directly to the mesophyll cells (Munné-Bosch et al., 1999), allowing photosynthesis to recover. In addition to supplying water to mesophyll cells, foliar water uptake also directs water to the xylem (Laur and Hacke, 2014), helping to repair air-filled conduits. The peltate scales on the abaxial surface in *P. polypodioides* function like wicks to direct and absorb water during foliar water uptake (Pessin, 1924; Potts and Penfound, 1948; John and Hasenstein, 2017, 2018), and our data using LY tracing dye corroborates this previous work showing that the peltate scales absorb water (Figure 8). The peltate scales function similarly to leaf trichomes that absorb water in angiosperm species (Franke, 1967; Benzing et al., 1978; Schreel et al., 2020). Furthermore, this even distribution of a surface film of water facilitated by the peltate scales would prevent the leaf from transpiring and generating negative water potentials, thereby providing conditions that would facilitate embolism repair if the leaves remained saturated for long periods of time.

Foliar water uptake is an important water acquisition strategy for plants in communities that rely on dew, fog, mist, and cloud water for a substantial proportion of their water sources, such as tropical montane forests (Goldsmith et al., 2012) and temperate rainforest epiphytic communities (Limm et al., 2009), as well as in Mediterranean climates (Munné-Bosch et al., 1999) and more arid ecosystems (Cavallaro et al., 2020). In fact, foliar water uptake has been observed in most plant families across many major plant biomes (Berry et al., 2019). While foliar water uptake is widespread, it is particularly beneficial for epiphytic DT fern *P. polypodioides* that inhabit regions with high amounts of rainfall. By absorbing water through the leaves, *P. polypodioides* can recover photosynthetic capacity after desiccation without attachment to the rhizome or root system. While this unique ability of fronds to recover from desiccation without rhizome attachment was observed under artificial conditions, it highlights the durability of these epiphytic DT ferns and a shift from relying on root pressure and support from the rhizome for recovery. Once detached, the fronds do have a finite lifespan, but if kept in humid conditions these fronds can survive for an extended period of time (Supplemental Movie S2).

### Desiccation tolerance in epiphytic ferns

Based on our data from *P. polypodioides*, desiccation tolerance in epiphytic ferns may operate differently from desiccation tolerance in terrestrial, xeric ferns. Notably, the dependence on arboreal substrates with highly variable moisture regimes has perhaps favored the use of foliar water uptake for quick recovery. Furthermore, the highly vulnerable stipe xylem in this species—with a turgor loss point of −1.35 (±0.31 sd) MPa—suggests that cavitation in the stipe may act as a “hydraulic fuse” (Tyree et al., 1993; Wolfe et al., 2016), preventing excessively low water potentials to arise in the rhizome. By cutting off the evaporative surface area of the pinnae via cavitation in the stipe, the rhizome would conserve water until a future precipitation event that would allow for new growth. The fact that this species can continue to photosynthesize even after being disconnected from the rhizome (both physically in our excision experiment, but also hydraulically due to cavitation) could allow the frond to continue to produce spores and reproduce. Indeed, hydraulic disconnection may be necessary to induce dehydration in the leaf and dehiscence to disperse the spores upon maturity. Thus, instead of relying heavily on root pressure during desiccation recovery (Holmlund et al., 2020), this epiphytic fern appears to rely more on foliar water uptake, utilizing a strategy that makes it supremely capable of growing in a water-limited environment above ground. Aside from the potentially high prevalence of desiccation tolerance in the Hymenophyllaceae family, there are few epiphytic DT ferns (Porembski, 2011). The combination of being epiphytic and DT may require a special set of traits and environmental conditions, including periodic rainfall events, substrates that retain moisture, and the fern’s ability to absorb water through the leaves. As climate change makes rainfall more variable across parts of southern North America and Central America (Romero-Lankao et al., 2014), DT epiphytic ferns like *P. polypodioides* may have difficulty surviving long periods of desiccation.

## Conclusion

Here, we reveal the impact of desiccation and rehydration on the hydraulic and photosynthetic systems of epiphytic DT fern *P. polypodioides*. Evaporation from the leaf leads to rapid embolism accumulation in the stipe, declining water potentials, and loss of RWC and turgor. Frond photosynthetic rates and chlorophyll fluorescence decline in parallel. The hydraulic decoupling of the leaf and rhizome likely prevents highly negative water potentials from developing in the rhizome, thereby acting as a “hydraulic fuse” (Tyree et al., 1993; Wolfe et al., 2016). However, in contrast to previous work on the “hydraulic fuse” hypothesis, our data show that *P. polypodioides* fronds can persist despite the hydraulic decoupling due to their capacity for foliar water uptake. While cycles of dehydration and rehydration occur frequently in the field, periods immediately following precipitation events may be sufficient to meet the carbon demands of the individual leaves. Our microCT data show that the tissue within the endodermis, including the phloem, rehydrate before embolism repair in the tracheids, which suggests that the export of carbon from the leaf to the rhizome may be possible even without a functional hydraulic connection. A second advantage of the hydraulic decoupling would be for the purposes of reproduction. The frond would need to dehydrate to allow for the desiccation of the mature sori for spore dispersal. Ultimately, this strategy is ideal for an epiphyte growth in regions that receive frequent precipitation, as fronds are essentially in equilibrium with their environments and rely on frequent rainfall and foliar water uptake to survive desiccation events. Ferns are an evolutionary stepping stone between nonvascular plants and seed plants (López-Pozo et al., 2018), thus our understanding of resurrection dynamics in both terrestrial and epiphytic ferns can help to further our understanding of differences in resurrection dynamics across the plant phylogeny.

## Materials and methods

### Study species

*Pleopeltis polypodioides* is an epiphytic, sometimes rupestral, resurrection fern in the Polypodiaceae family. It often grows on the branches of large trees, particularly live oaks (Moran, 2004). It is distributed from eastern North America to South America, and is particularly widespread in Central America (GBIF, 2019).

One-inch rhizome sections with fronds of *P. polypodioides* were grown in the Greeley greenhouse at Yale University and subject to 20 s of misting every 30 min to maintain a well-hydrated water status.

### Brightfield microscopy

To aid in the interpretation of the grayscale X-ray microCT images of the stipe, we made freehand transverse cross sections of the stipe under both well hydrated (Figure 1;Supplemental Figure S1A) and dehydrated (Supplemental Figure S1B) conditions. These sections were made using a razor blade from the base of the stipe in approximately the same location where the microCT imaging was performed. Sections were first immersed in a 10% v/v bleach solution to clear the tissue and remove phenolic compounds, and then rinsed in deionized water. Next the sections were stained with a combination of Safranin and Astrablue (Gärtner and Schweingruber, 2013), and then rinsed again in deionized water. Finally, sections were mounted in water on a slide and imaged with a 20× magnification objective on a compound microscope (Olympus BX60, Olympus America, Center Valley, PA, USA) attached to a Canon 6D digital SLR camera (Canon, Melville, NY, USA). Unstained freehand transverse sections were made from the base of the stipe on dehydrated frond material (Supplemental Figure S1B). Dehydrated cross sections were not mounted in water to preserve the dehydrated state of the sections.

### Characterizing dehydration

Because all of our treatments simulated droughts through dehydration events, we characterized the severity of these dehydrations by measuring RWCs and water potentials over time, as well as by measuring gas exchange while fronds dehydrated (mean temperature of 24.1°C, mean relative humidity of 29.7%, and mean PPFD 5 µmol (photons) m^−2^ s^−1^ (Hobo Logger MX1104, Onset Computer Corp., Bourne, MA, USA). For RWC, fronds attached to their one-inch rhizome sections were weighed every 10 s during a 24-h dehydration to calculate the RWC of the plants. We used fronds attached to rhizome sections in order to maintain consistency between our experiments, instead of measuring RWC separately on frond and rhizome material. Prior to beginning the dehydration, each plant was allowed to reach a turgid state by floating frond and rhizome material in petri dishes of water for 2 h. The turgid weight of the plant material was then recorded on a Sartorius balance (Practum 224-1s, Sartorius, Gottingen, Germany), and the balance was configured to record the weight of the plant material every 10 s using WinWedge software (TAL Technologies Inc., Philadelphia, PA, USA). After 24 h of dehydration, each plant was removed from the balance and oven-dried at 80°C for 24 h to record the oven-dried weight. This process was repeated for five plants. RWC for all plants was calculated as RWC % = [(Weight – Dry Weight)/(Turgid Weight – Dry Weight)]*100.

In addition to measuring RWC throughout a 24-h dehydration, we measured rhizome water potentials on a separate group of eight fronds during dehydration using stem psychrometers (ICT International, Armidale, NSW, Australia). To install the stem psychrometers on the rhizome, the outer layer of the rhizome was gently scraped away as close to the base of the frond as possible to expose the xylem and allow the thermocouple of the psychrometer to be installed. Psychrometers were then wrapped in parafilm around the rhizome to maintain connection to the plant; given that rhizomes were tightly sealed with parafilm to secure the psychrometers, the rate of rhizome water potential decline during dehydration is likely a conservative estimate. These psychrometers recorded water potentials every 10 min during a 24-h dehydration. After 24 h, the fronds were rehydrated by wrapping the fronds in damp paper towels and enclosing them in plastic bags.

Pressure-volume curves were conducted on fronds (*n* = 6) using a Scholander pressure chamber (Model 600, PMS Instruments, Albany, OR, USA) and following established protocols for bench drying methods from the literature and Prometheuswiki (Tyree and Hammel, 1972; Koide et al., 2000; Sack et al., 2003; Sack and Pasquet-Kok, 2011). For the initial rehydration, fronds were wrapped in damp paper towels and enclosed in plastic bags. Fronds were cut with a razor blade at the base of the stipe and put in Whirl-Pak bags (Whirl-Pak, Madison, WI, USA) before measuring water potential, and then were immediately weighed on a Sartorius balance (Practum 224-1s, Sartorius, Gottingen, Germany). This process was repeated as fronds were bench dried until they reached a water potential of about −2.1 to −3.0 MPa. Then, the fronds were oven-dried at 80°C for 36 h to record the dry weight.

We measured gas exchange on fronds (*n* = 3) to observe stomatal conductance and photosynthetic rate during a 6-h dehydration (LI-6400 equipped with the conifer chamber [6400-05; Li-Cor, Lincoln, NE, USA]). For each frond, the chamber was set to maintain the following conditions: 400 ppm CO_2_, 50% RH, and 300 µmol (photons) m^−2^ s^−1^ PPFD (external light; LumiGrow Pro 325 LD, Emeryville, CA, USA). Fronds were kept well hydrated with damp paper towels wrapped around the leaf and rhizome tissue until the start of measurements. Next, the paper towels were removed, and we then recorded gas exchange every 30 s over the 6-h period as the fronds dehydrated.

### Visualizing dehydration and rehydration: frond-only and rhizome-only rehydration

A frond attached to a 2.5-cm rhizome segment was monitored for detailed observations of the physical changes to the leaf tissue during dehydration and rehydration. The rhizome was mounted in a clamp and positioned inside a thin plastic clamshell container covered in plastic wrap with a hole large enough to accommodate the camera lens cut in the side. The chamber was constructed to maintain high humidity during the observation period. Pieces of tissue paper were positioned on either side of the frond but outside the field of view of the camera. A datalogger (Hobo MX1101; Onset Computer Corp., Bourne, MA, USA) was also placed inside the chamber to monitor air temperature and relative humidity every 30 min. A piece of thick black cardstock was positioned in the background ∼2.5 cm from the frond. We photographed the frond every minute for ∼5 d with a digital camera (Canon RP with a 100 mm f = 2.8 lens) and constant illumination from an LED light source (MC RGBWW, Aperture Imaging Industries, Los Angeles, CA, USA). The frond began the experiment in the curled, dehydrated state. The leaf was then periodically sprayed with water to rehydrate the leaf while maintaining the relative humidity at ∼100% until the leaf had fully rehydrated. The leaf was then allowed to dry at ambient conditions as the relative humidity declined and reached equilibrium with the surrounding air. The image sequence was then assembled into a time-lapse movie using Adobe Premiere Pro (Adobe Systems, San Jose, CA, USA). The temperature and relative humidity data were then added to the time-lapse video (Supplemental Movie S1).

We allowed a separate group of fronds attached to their one-inch rhizome sections to undergo benchtop dehydration under ambient laboratory conditions (mean temperature 23°C, mean relative humidity 40%, and mean PPFD 5 µmol (photons) m^−2^ s^−1^) for 43 h (*n* = 5). After dehydration, we detached fronds from the rhizome and placed damp paper towels underneath the abaxial leaf surface in order to continually supply water to the leaf tissue. To avoid the damp paper towels from drying out, we also applied water to the leaf surface using a water dropper every hour during rehydration. These fronds were positioned under a time-lapse camera (TimelapseCam Pro, Wingscapes, Calera, AL, USA) and photographed hourly for a few weeks to document the physical changes of the frond during dehydration and track whether recovery of the fronds was possible without attachment to the rhizome but with the addition of foliar water. Twenty-four hours after beginning open-air rehydration, we placed the fronds inside 50-mL plastic centrifuge tubes with a wet paper towel to determine how long these detached fronds could survive in the continued presence of moisture without connection to the rhizome. Time-lapse imaging continued for a month to document the state of the detached fronds during rehydration (Supplemental Movie S2).

After rehydrating only the fronds, we performed a similar experiment without detaching fronds and with only rehydrating the rhizome sections. Fronds and their rhizome sections underwent benchtop dehydration under ambient laboratory conditions (mean temperature of 24.1°C, mean relative humidity of 29.7%, and mean PPFD 5 µmol (photons) m^−2^ s^−1^) for 24 h (*n* = 4). After 24 h of dehydration, the rhizomes were placed in petri dishes of water, carefully positioned so that the leaf material did not get wet. The time-lapse camera (TimelapseCam Pro; Wingscapes, Calera, AL, USA) photographed the fronds hourly for 36 h to track their rehydration via the rhizome (Supplemental Movie S3).

### Visualizing dehydration and rehydration: microCT

Fronds attached to one-inch rhizome sections were shipped in sealed 50-mL plastic centrifuge tubes—with ample water droplets in the tubes and on the plants to keep the material hydrated—to the X-ray micro-computed tomography facility (beamline 8.3.2) at the Lawrence Berkeley National Laboratory Advanced Light Source (ALS) in Berkeley, California. Prior to scanning, we captured an image of the spatial distribution of chlorophyll fluorescence (*F_v_′/F_m_′*) on the abaxial side of 12 fronds with an IMAGING PAM M-Series MINI fluorometer (Walz, Effeltrich, Germany; see Chlorophyll fluorescence imaging methods below). We then divided these 12 fronds into four experimental groups that would undergo different levels of dehydration (Supplemental Table S1). Group A was a well-hydrated control group that was not allowed to dehydrate during the experiment. Group B was allowed to dehydrate on the bench at ambient conditions for 3 h (mean temperature 21°C, mean relative humidity 70%, mean PPFD ∼0 µmol (photons) m^−2^ s^−1^). Group C was dehydrated for 12 h, and Group D was dehydrated for 24 h, both under the same ambient bench conditions. We prepared the centrifuge tubes so they could be mounted in the microCT sample holder by drilling a hole through the bottom and attaching a screw that could be fixed and mounted in the X-ray beam. Fronds and their rhizome sections were then mounted in these tubes between two pieces of polystyrene foam to keep the stipe centered and immobile during the scanning period. Stipes were scanned at 15 keV using the 10× magnification and continuous tomography setting on the instrument, where the stipe was rotated in 0.125° increments over 180° following previous X-ray tomography methods (Brodersen et al., 2011, 2012; McElrone et al., 2013). Scans were completed in ∼10–15 min, and the raw images were reconstructed using TomoPy (Gürsoy et al., 2014), a Python-based framework for reconstructing tomographic images. The final pixel resolution of the images is 0.65 µm.

After we scanned each dehydrated group, we rehydrated the fronds by applying water with a water dropper to both the rhizome and fronds until the plants were covered in a film of water. Rhizome and frond sections were then enclosed in their centrifuge tubes to maintain a humid environment for rehydration. We allowed the ferns to recover for the same amount of time that they were dehydrated (i.e. 3 h of dehydration followed by 3 h of rehydration for Group B, etc.). We then repeated fluorescence imaging, and each frond in each group was scanned again in the rehydrated state. Finally, we scanned a few well-hydrated and dehydrated leaves (*n* = 4) to determine the hydration status of the leaf mesophyll.

### Chlorophyll fluorescence imaging

An IMAGING PAM M-Series MINI fluorometer (Walz, Effeltrich, Germany) was used to capture images of the *F_v_′/F_m_′*—the ratio of light-adapted variable and maximal fluorescence—of abaxial frond tissue prior to each microCT scan at the beamline. Fronds were subjected to a saturating light pulse and imaged with the fluorometer camera. The software then calculated the *F_v_′/F_m_′* of each pixel of leaf tissue in the image and applied a false-color scale to the images. We took baseline images of abaxial leaf surfaces of each frond while they were well-hydrated, and then took images of the fronds in each Group (A–D) while they were dehydrated and then rehydrated.

Additional fluorescence imaging was conducted separately from the microCT experiments; in this separate imaging, the adaxial and the abaxial *F_v_′/F_m_′* values of five fronds were compared in both well-hydrated and dehydrated states (24 h of dehydration). Since leaf curling occurs during dehydration, the pinnae were carefully flattened to expose the adaxial leaf surface during the 24-h dehydrated images. All fronds, whether well-hydrated or dehydrated, were imaged underneath clear plastic wrap that was clipped to a piece of cardboard in order to flatten the pinnae for imaging. Additional images were taken on five well-hydrated fronds from which we removed peltate scales from the abaxial leaf surface to determine if the presence of scales impacted the *F_v_′/F_m_′* values of abaxial leaf tissue.

The ImagingWinGigE software that accompanies the IMAGING PAM M-Series MINI fluorometer (Walz, Effeltrich, Germany) was used to analyze the fluorescence images. We measured the *F_v_′/F_m_′* of the leaf tissue by creating 10 circular areas of interest (AOI), where the *F_v_′/F_m_′* of each pixel was calculated and averaged for that AOI. The mean of these 10 AOIs was taken to produce a mean *F_v_′/F_m_′* for that image. In some of the images both the adaxial and abaxial leaf surfaces were visible due to leaf curling during dehydration; separate AOIs and mean values were calculated for abaxial and adaxial leaf surfaces.

### Image processing: ImageJ & Avizo

The raw tomographic projection images from the microCT were reconstructed to stacks of 2,160 2D images using python programming. Once we obtained the reconstruction image stacks, we used ImageJ (version 2.35, US National Institutes of Health, Bethesda, Maryland, USA) for analysis of xylem and stipe anatomy. Specifically, we measured the cross sectional xylem area, embolized xylem area, stele area and perimeter, the lacunae gap area between the cortex and the stele, tracheid diameters, and whole stipe area on the center slice of each reconstruction stack (similar to parameters measured in (Holmlund et al., 2019)). To measure areas, an outline was drawn around the anatomical region of interest. The percent of embolized xylem area was then calculated based on the total xylem area. If multiple bundles were present within the stipe, we simply summed the area of each measured anatomical parameter. For tracheid diameter measurements, we measured all of the tracheids in nine of the scans (*n* = 741 tracheids) to obtain an accurate tracheid diameter distribution. We also examined our image stacks in Aviso software (FEI, Hillsboro, OR, USA) using 2D and 3D visualizations.

### Frond detachment and photosynthetic recovery

To determine the role of attachment to and detachment from the rhizome during dehydration and rehydration we measured photosynthesis using an LI-6400 equipped with the conifer chamber (6400-05; Li-Cor, Lincoln, NE, USA) on four groups of three fronds. The chamber conditions were set to 400 ppm CO_2_, 50% RH, and 300 µmol (photons) m^−2^ s^−1^ PPFD (external light, LumiGrow Pro 325 LD, Emeryville, CA, USA). A hydrated control group was kept well hydrated for the duration of the experiment (Supplemental Table S1). An attached group was dehydrated for 24 h and rehydrated for 24 h while fronds remained attached to their one-inch rhizome sections. Similarly, a detached group underwent the same 24 h of dehydration followed by 24 h of rehydration, but fronds were detached from rhizomes after the initial baseline measurements. Lastly, a second detached group was dehydrated for the duration of the experiment, without any rehydration (Detached and n.r; *n* = 3 per group). Hydration was maintained for the hydrated control group by wrapping rhizomes or stipes ends in damp towels and gently placing them in 50-mL plastic centrifuge tubes. These tubes were placed in sealed plastic bags and the leaf material that remained outside of the tubes was wrapped in damp towels to create a humid microenvironment. This process was repeated for the attached and detached groups upon their rehydration.

Prior to measurements, all 12 fronds were photographed for leaf area calculation in ImageJ (version 2.0, US National Institutes of Health, Bethesda, Maryland, USA) to recalculate the photosynthetic measurements based on the leaf area inside the conifer chamber. If applicable, fronds were patted dry with a paper towel to remove any surface film that would inhibit transpiration before being enclosed in the chamber. Fronds were enclosed in the chamber just below the most basal pinnae, and allowed to equilibrate for 10–15 min before logging three measurements per frond. Fronds were measured at the beginning of the experiment prior to any dehydration or detachment from the rhizome to get a baseline photosynthetic rate under well-hydrated conditions. After baseline measurements were taken, we dehydrated fronds from the attached group and both detached groups, and detached fronds from the rhizome for both detached groups. Photosynthetic measurements were then repeated for each group at 3, 12, 16, 20, and 24 h. After 24 h, the attached group and one detached group were rehydrated, and measurements were repeated at 27, 35, 39, 43, and 47 h.

### Foliar water uptake and fluorescence microscopy

We used the foliar water uptake tracer, LY, diluted to 1 mM v/v (Thermo Fisher Scientific, Waltham, MA, USA; Schreel et al., 2020) on the abaxial surface of the leaf to determine the potential for water uptake across the epidermis via the peltate scales. Six leaves were collected and placed on the laboratory bench under similar conditions as the other dry-down experiments. After 15 min, a drop of dye was placed on the abaxial surface of each leaf for 15 min, and then the excess dye was blotted off. Surface images of the leaves were then taken with Canon 6D digital SLR camera (Canon, Melville, NY, USA) attached to a compound microscope (Olympus BX60; Olympus America, Center Valley, PA, USA) equipped with a filter cube for LY fluorescence (excitation 520–550 nm, emission 570–650 nm). Images were collected from regions where the dye drop was placed, as well as adjacent undyed regions to compare the presence or absence of LY fluorescence. Transverse leaf sections were made 30 min after the application of the dye and imaged. We collected multiple images at each position while changing the focal plane to create a stack of images which were then merged together in Adobe Photoshop CS (Adobe Systems, San Jose, CA, USA).

### Statistics

All statistics were conducted within R version 3.6.2 (R Foundation for Statistical Computing, Vienna). Repeated measures ANOVAs were calculated for percent xylem area embolized, *F_v_′/F_m_′*, and photosynthetic measurements using the “rstatix” package (version 6.0, Kassambara).

## Supplemental data

The following materials are available in the online version of this article.

**Supplemental Figure S1.** Light micrographs of freehand stipe transverse sections of *P. polypodioides* when hydrated and dehydrated.

**Supplemental Figure S2.** X-ray microCT images of well-hydrated and dehydrated pinnae.

**Supplemental Figure S3.** Tracheid diameter distribution and diameters of embolized and nonembolized tracheids.

**Supplemental Figure S4.** Representative abaxial and adaxial *Fv′/Fm′* images for well-hydrated and dehydrated states, and differences in abaxial and adaxial *Fv′/Fm′* with or without peltate scales.

**Supplemental Table S1.** Experimental groupings and the conditions for each group.

**Supplemental Movie S1.** Dehydration and rehydration of a *P. polypodioides* frond with concurrent relative humidity (blue line) and temperature (red line) measurements.

**Supplemental Movie S2.** Time-lapse movie of frond-only rehydration.

**Supplemental Movie S3.** Time-lapse movie of rhizome-only rehydration.

## Supplementary Material

kiab361_Supplementary_Data

## Notes

C.B. came up with the original idea, and K.P. and C.B. designed the study. K.P. performed the experiments and collected the data. K.P. and C.B. analyzed the data and completed the writing.

The author responsible for distribution of materials integral to the findings presented in this article in accordance with the policy described in the Instructions for Authors (https://academic.oup.com/plphys/pages/General-Instructions) is Kyra Prats (kyra.prats@yale.edu).
