# Supplementary material for: Desiccation and rehydration dynamics in the epiphytic resurrection fern Pleopeltis polypodioides
Source: Plant Physiol. 2021 Aug 2;187(3):1501–18. doi: 10.1093/plphys/kiab361 (PMC8566288; doi:10.1093/plphys/kiab361)
Supplement: kiab361_Supplementary_Data [file kiab361_supplementary_data.zip › Supplemental_Prats_PlantPhys21.docx]

**SUPPLEMENT**

**TABLES**

**Table S1.** Experimental groupings and the conditions for each group

| **Experiment** | **Groups** | | | |
| --- | --- | --- | --- | --- |
| **Xylem status (microCT scans)** | **Group A** | **Group B** | **Group C** | **Group D** |
|  | Hydrated control group (attached to the rhizome) | 3 hour group (attached to the rhizome) | 12 hour group (attached to the rhizome) | 24 hour group (attached to the rhizome) |
|  | Scanned twice | Scanned at 3 hours dehydrated and 3 hours rehydrated | Scanned at 12 hours dehydrated and 12 hours rehydrated | Scanned at 24 hours dehydrated and 24 hours rehydrated |
| ***F_v_’/F_m_’*** | **Group A** | **Group B** | **Group C** | **Group D** |
|  | Hydrated control group (attached to the rhizome) | 3 hour group (attached to the rhizome) | 12 hour group (attached to the rhizome) | 24 hour group (attached to the rhizome) |
|  | Imaged prior to both well-hydrated scans | Imaged while well-hydrated and then prior to both dehydrated and rehydrated scans | Imaged while well-hydrated and then prior to both dehydrated and rehydrated scans | Imaged while well-hydrated and then prior to both dehydrated and rehydrated scans |
| **Photosynthesis** | **Hydrated** | **Attached** | **Detached** | **Detached (n.r.)** |
|  | Hydrated control group attached to the rhizome | Attached to rhizome group | Detached from rhizome group | Detached and not rehydrated (n.r.) group |
|  | Measurements taken at all timepoints | Dehydrated for 24 hours, rehydrated for 24 hours | Detached from rhizome at the start;  Dehydrated for 24 hours, rehydrated for 24 hours | Detached from rhizome at the start;  Measurements taken at all timepoints;  Not rehydrated |

**FIGURES**


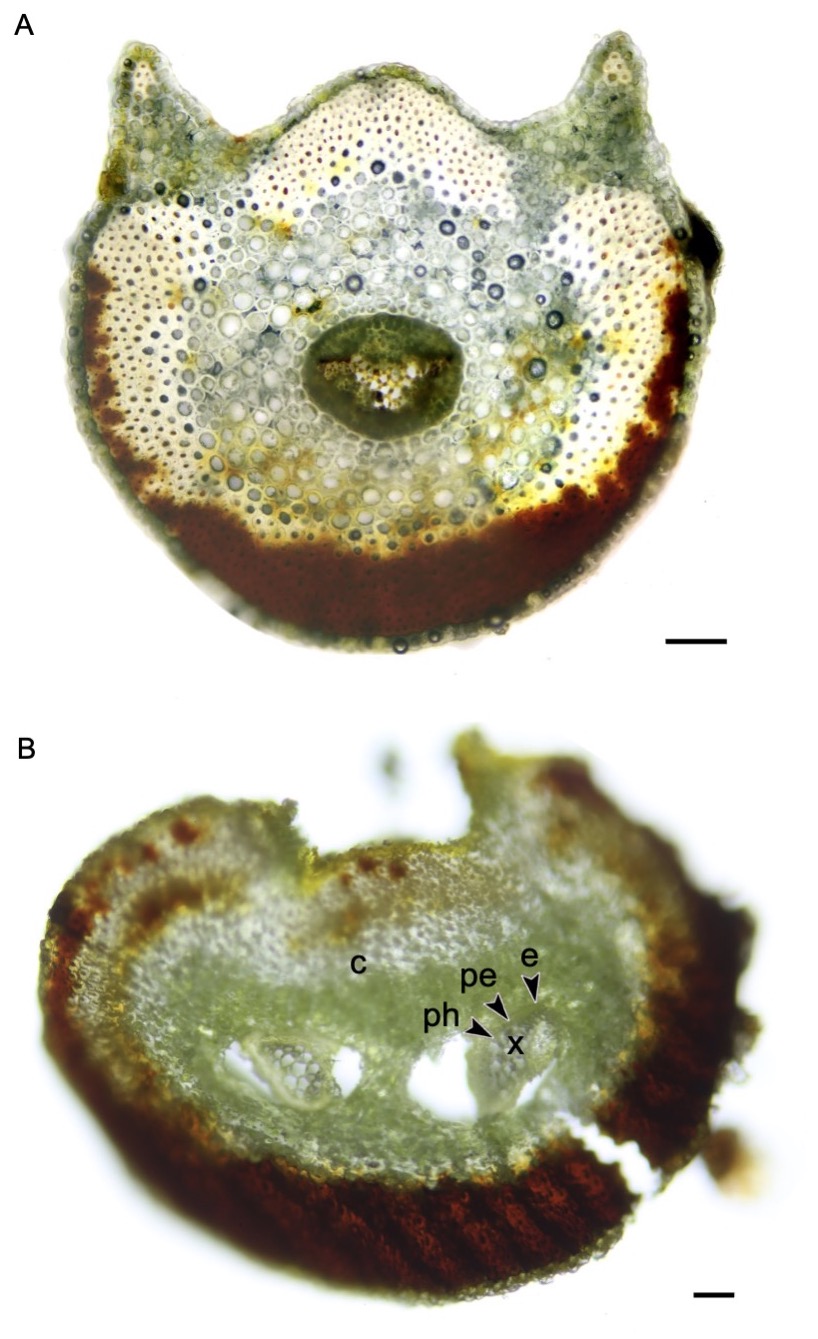


**Supplemental Figure S1. Light micrographs of freehand stipe transverse sections of *P. polypodioides* when hydrated and dehydrated.** A) Freehand transverse section of an unstained and hydrated stipe. B) The dehydrated transverse section shows that the separation of the vascular bundles occurs between the endodermis (e) and the surrounding cortex cells (c). Most of the cell collapse likely occurs in the pericycle (pe) tissue just inside the endodermis, and perhaps some of the phloem (ph). The xylem (x)—which is much more rigid—does not collapse during the dehydration process. The dehydrated cross section was mounted on the slide without water or glycerol to not rehydrate the material. All scale bars = 0.01mm.


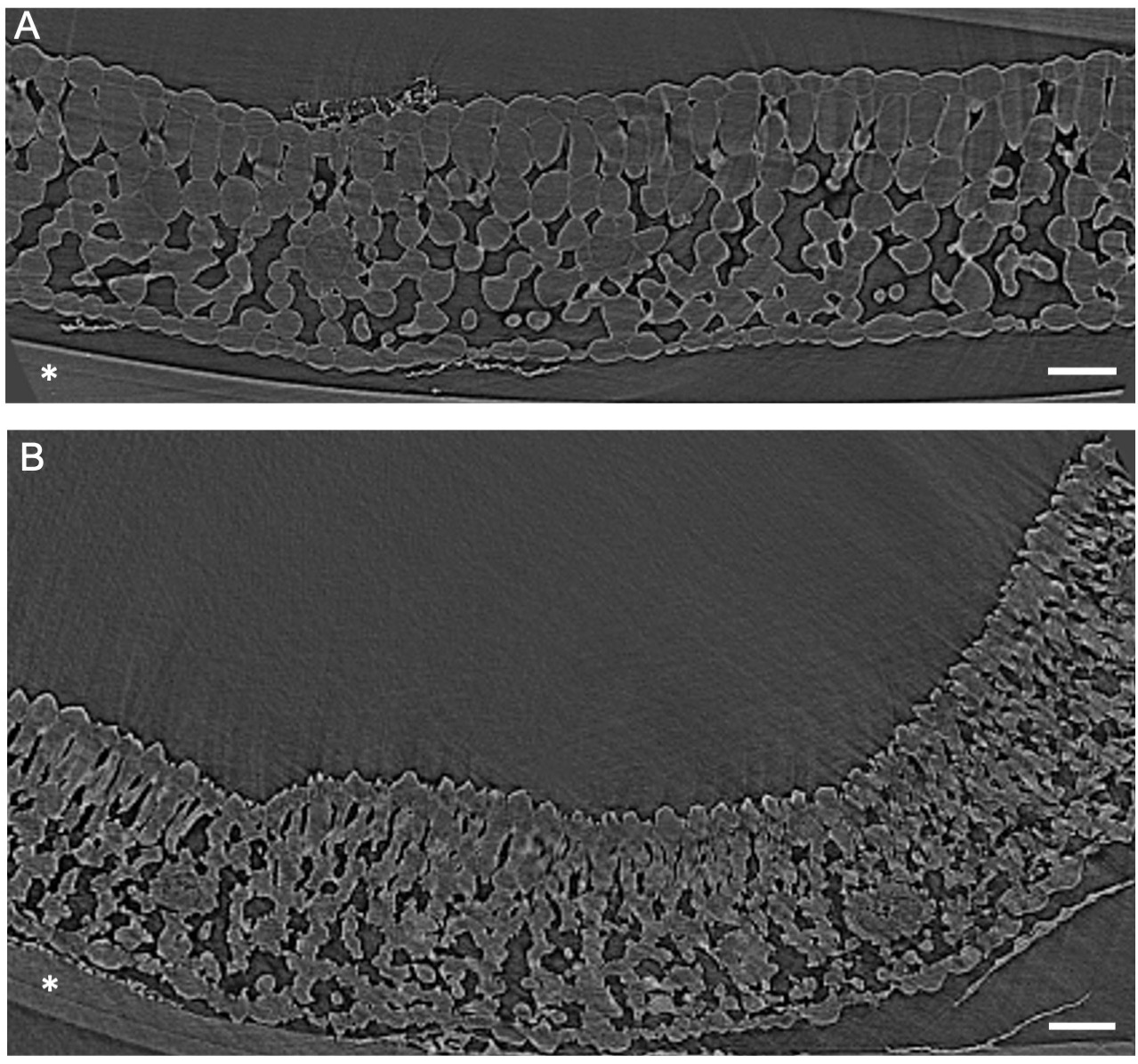


**Supplemental Figure S2. X-ray microCT images of well-hydrated and dehydrated pinnae.** A) The well-hydrated pinna, with fully hydrated mesophyll cells. B) The dehydrated pinna experienced 6 hours of dehydration. The mesophyll cells in the dehydrated pinna appear visibly shrunken. Both pinna in A) and B) are oriented with the adaxial side of the leaf tissue facing towards the top of the images. Pieces of tape were used to hold leaf sections in place during the scan, and are marked with asterisks. All scale bars = 0.1mm.

**Supplemental Figure S3. Tracheid diameter distribution and diameters of embolized and non-embolized tracheids.** A) Tracheid diameter distribution; the mean tracheid diameter was 7.8 μm (± 4.1 SD, n = 741 tracheids), marked by the vertical gray dashed line. B) The t-test between the mean diameter of non-embolized (N, n = 26) and embolized (Y, n = 251) tracheids at 3 hours dehydrated was significant (t = -4.3, df = 43.6, P = 0.00009, ***). C) Similarly, the t-test between the mean diameter of non-embolized (N, n = 254) and embolized (Y, n = 21) tracheids at 24 hours rehydrated was significant (t = -6.6, df = 21.8, P = 0.000001, ***). In B) and C) the triangles represent the mean, the center lines are the median, the box limits are the upper and lower quartiles, the whiskers are 1.5x interquartile range, and the points are the outliers. It is important to note that the tracheid counts for both tests were not evenly distributed.


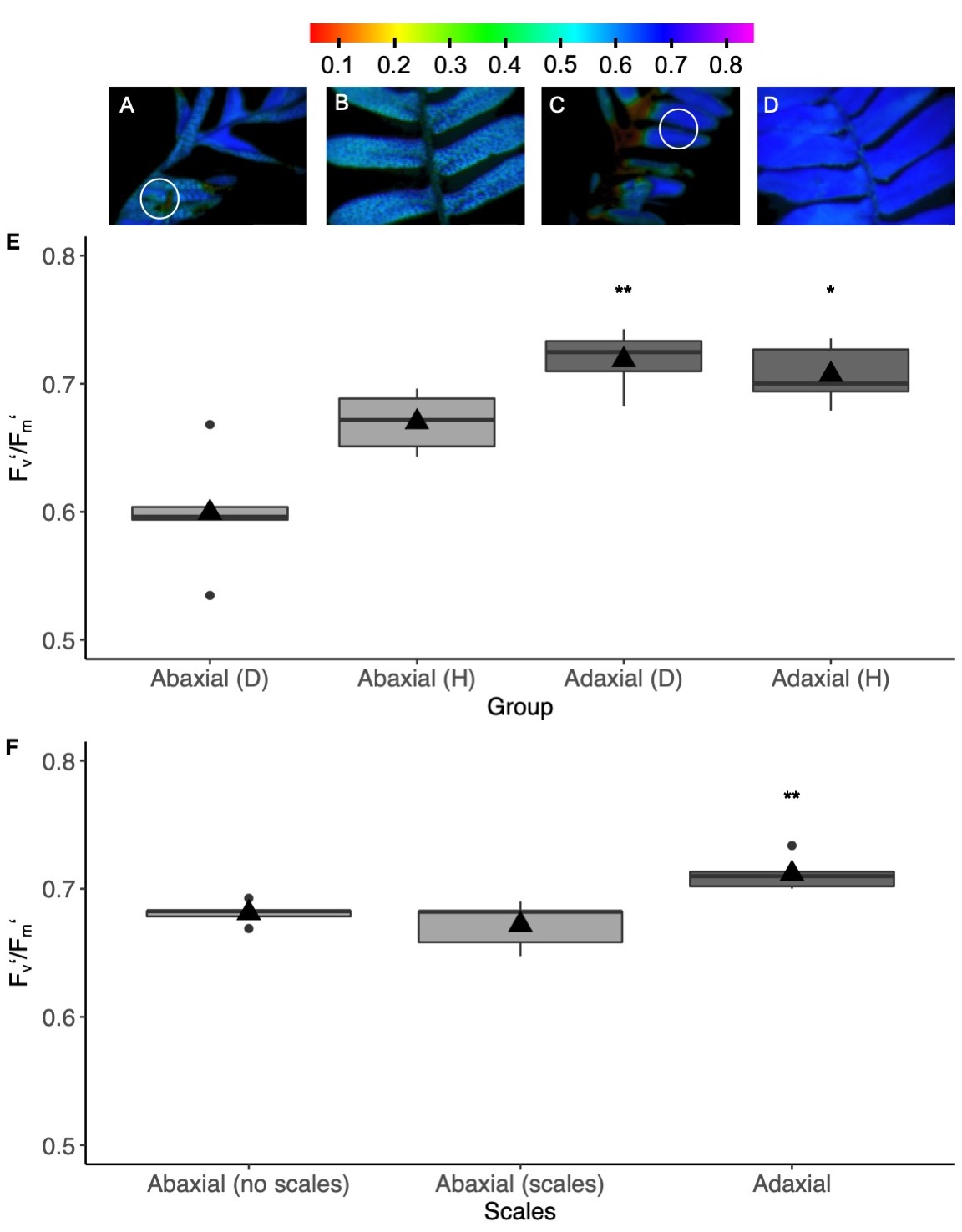


**Supplemental Figure S4. Representative abaxial and adaxial *Fv’/Fm’* images for well-hydrated and dehydrated states, and differences in abaxial and adaxial *Fv’/Fm’* with or without peltate scales.** Images show the A) abaxial dehydrated, B) abaxial hydrated, C) adaxial dehydrated, and D) adaxial hydrated examples. The circles in A) and C) show examples of leaf tissue that was used for the measurements in cases where abaxial and adaxial leaf tissue were both present. All scale bars = 5 mm. E) The abaxial (light gray) and adaxial (dark gray) *Fv’/Fm’* was significantly different in both hydrated (H) and dehydrated (D) states (ANOVA, F(3, 16) = 14.9, P < 0.0001). Furthermore, post-hoc analyses with a Bonferroni adjustment confirmed that the abaxial and adaxial *Fv’/Fm’* was significantly different while well-hydrated (P = 0.038, *) as well as after 24 hours of dehydration (P = 0.002, **). F) The removal of peltate scales from the abaxial surface did not cause any difference between abaxial *Fv’/Fm’* values with scales, and the adaxial (dark gray) *Fv’/Fm’* was significantly different from both abaxial (light gray) mean values (ANOVA, F = 11.16, P = 0.002, **). In E) and F) the triangles represent the mean, the center lines are the median, the box limits are the upper and lower quartiles, the whiskers are 1.5x interquartile range, and the points are the outliers

**MOVIES**


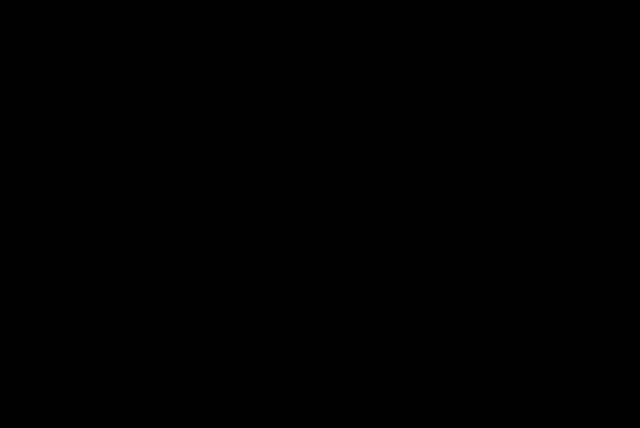


**Supplemental Movie S1**. **Dehydration and rehydration of a *P.* *polypodioides* frond with concurrent relative humidity (blue line) and temperature (red line) measurements.** Time-lapse images were collected every minute for approximately five days with supplemental misting of the leaf and rhizome.


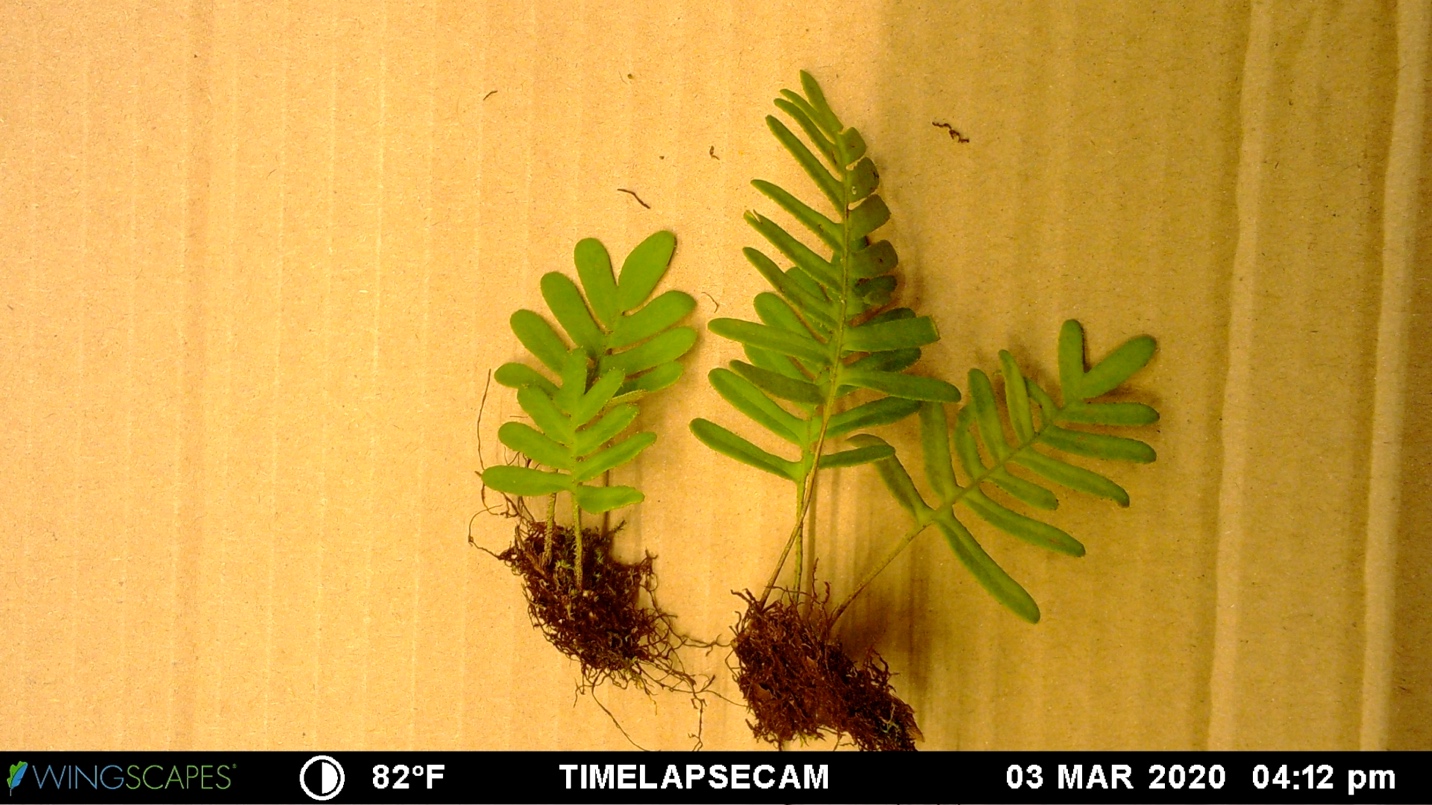


**Supplemental Movie S2**. **Time-lapse movie of frond-only rehydration**. Fronds were dehydrated for 43 hours starting the afternoon of March 3^rd^, 2020. Fronds were then detached from the rhizome and allowed to rehydrate, at first in open-air benchtop conditions, and then after 24 hours of rehydration the fronds were loosely enclosed in a centrifuge tube with a wet paper towel. Time-lapse images continued until April 16^th^, 2020 (images between March 7 and April 16 were cut for movie time). While time-lapse images stopped after a month and a half, the detached fronds remained green in the centrifuge tube for a total of 3 months (until July 2020).


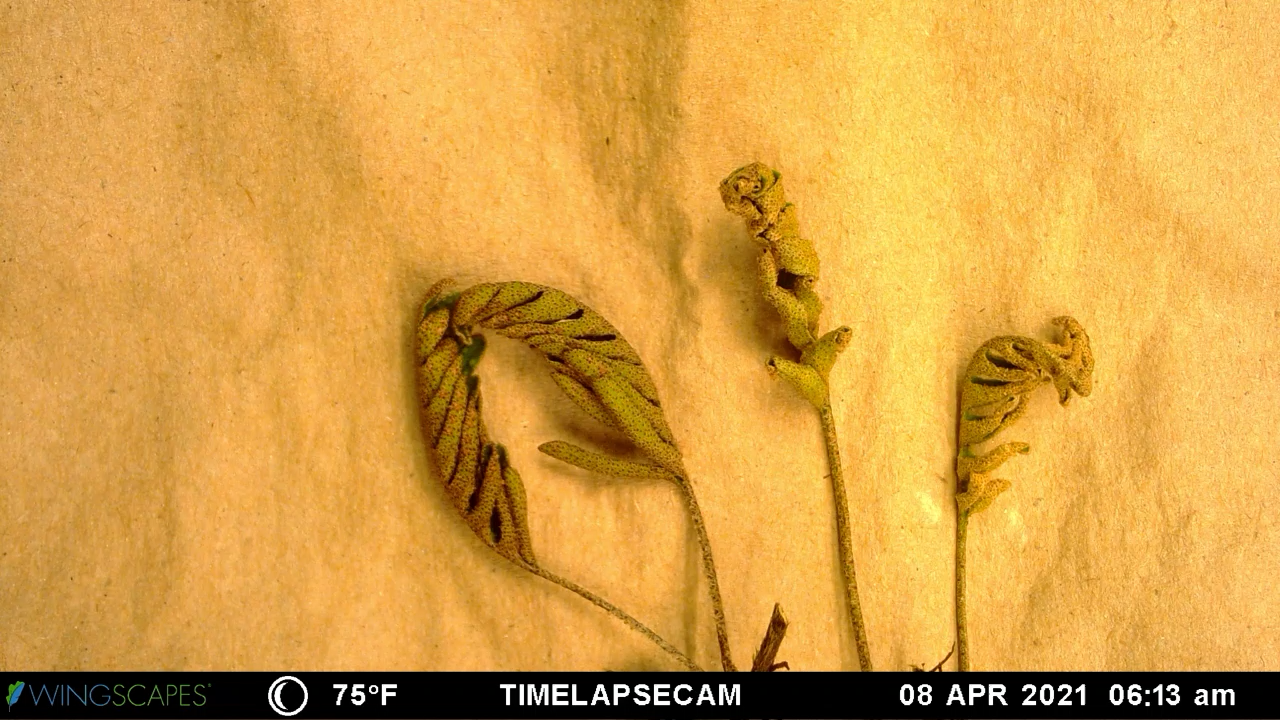


**Supplemental Movie S3.** **Time-lapse movie of rhizome-only rehydration.** Fronds and rhizomes were dehydrated for 24 hours in open-air benchtop conditions and then only the rhizome was supplied with water and rehydrated for 36 hours.
